# Dopamine in Idiopathic Polymorphic Ventricular Tachycardia/Ventricular Fibrillation

**DOI:** 10.19102/icrm.2021.120908

**Published:** 2021-09-15

**Authors:** Hussein Rabah, Zaynab Khalaf, Ali Rabah

**Affiliations:** ^1^Department of Internal Medicine, Staten Island University Hospital, New York, NY, USA; ^2^Department of Internal Medicine, Faculty of Medical Sciences, Lebanese University, Al Hadath, Lebanon; ^3^Division of Electrophysiology, Beirut Cardiac Institute (BCI), Beirut, Lebanon

**Keywords:** Dopamine, idiopathic ventricular fibrillation, polymorphic ventricular tachycardia

## Abstract

The role of medical therapy in the treatment of idiopathic polymorphic ventricular tachycardia (IPMVT) and idiopathic ventricular fibrillation (IVF) is not well established. Current medications in use include amiodarone, lidocaine, isoproterenol, verapamil, and quinidine. However, the use of dopamine for controlling such arrhythmias has never been described. We present an interesting case of IPMVT/IVF storm induced by short-coupled premature ventricular contractions. The arrhythmia was terminated acutely using dopamine infusion and was suppressed chronically using verapamil.

## Case presentation

A 53-year-old female patient was admitted to the hospital for syncope. The patient had a negative medical, surgical, and family history.

**Figure 1:**
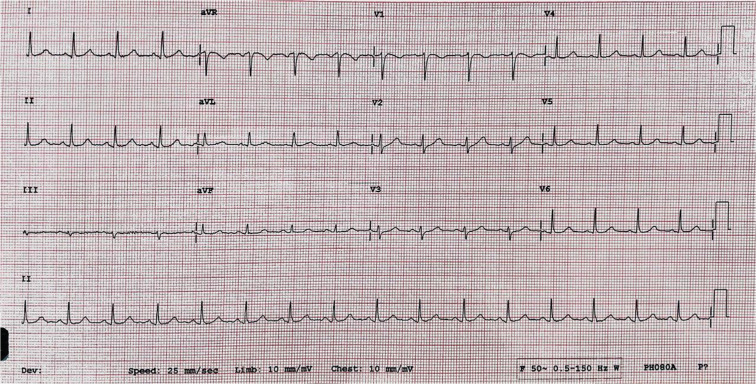
ECG recorded in the emergency room: normal sinus rhythm, a heart rate of 100 bpm, and a corrected QT of 415 ms. No ischemic changes or Brugada pattern were noted.

Assessment in the emergency department showed a hemodynamically stable patient with a pulse of 100 bpm and a blood pressure of 112/54 mmHg. Her physical examination was unremarkable. Sinus rhythm was evident on the electrocardiogram (ECG) **(Figure 1)**. Troponins; blood count; and blood levels of electrolytes, calcium, magnesium, and phosphorus were normal.

**Figure 2:**
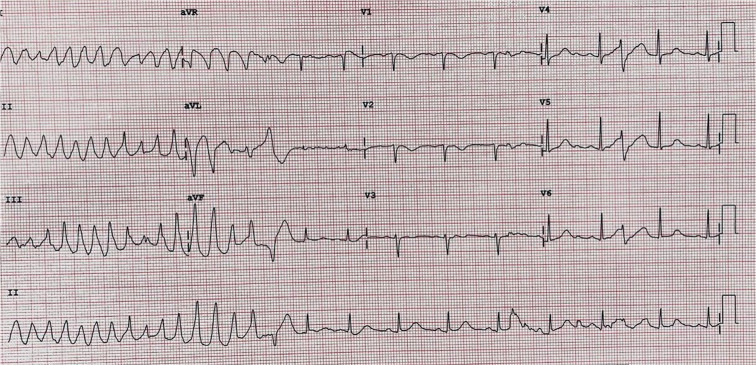
ECG showing PMVT.

The patient was admitted to the cardiac telemetry floor for further observation and management, where she was found to have polymorphic ventricular tachycardia (PMVT) **(Figure 2)** degenerating into ventricular fibrillation (VF) requiring electrical cardioversion. Amiodarone drip was started, followed by lidocaine drip as the PMVT/VF was recurrent, requiring multiple shocks.

**Figure 3:**
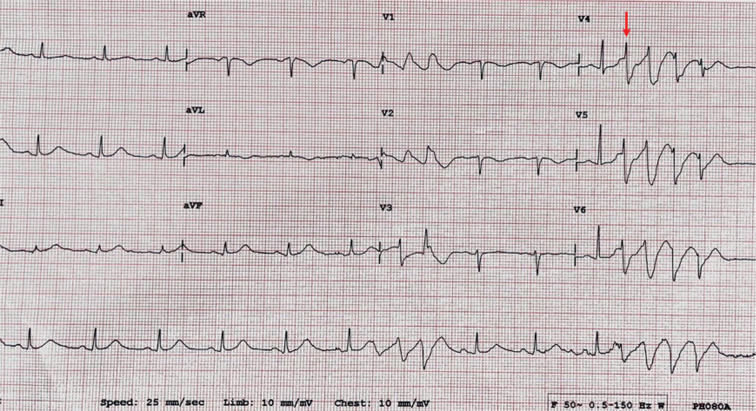
ECG showing a PVC (a coupling interval of 300 ms) initiating PMVT.

Due to refractory VF storm, the patient was sedated and intubated. Urgent coronary angiography showed no significant obstructive coronary artery disease. Furthermore, no structural heart abnormalities were apparent on echocardiography, and the cardiac function was normal. The ECG was remarkable for repetitive short-coupled premature ventricular contractions (PVCs) with a coupling interval of 300 ms **(Figure 3)** inducing PMVT/VF **(Figure 2)**. No prolonged QT, short QT, Brugada, or early repolarization patterns were recognized **(Figure 1)**.

Therefore, a diagnosis of idiopathic VF (IVF) was made, and intravenous verapamil boluses were administered to terminate the arrhythmia. However, VF was still ongoing, and overdrive ventricular pacing through a temporary transvenous pacer at a rate of 120 bpm failed to suppress the PMVT/VF. A total of 20 shocks were delivered. Consequently, due to the lack of isoproterenol at that time, a dopamine drip was started and titrated up to a minimum target heart rate of 110 bpm, after which the PMVT/VF storm ceased, and no more shocks were required despite the ongoing PVCs. Dopamine was tapered over the next three days, and verapamil was started and administered through a nasogastric tube, which caused a significant drop in her PVC burden.

The patient was then extubated, and a dual-chamber defibrillator was implanted before discharging her home on 240 mg of oral verapamil daily. Eight months of follow-up showed no more PMVT/IVF episodes with scarce PVCs.

The patient was informed that the data concerning her case will be submitted for publication and approved and gave consent for publication.

## Discussion

In 1994, Leenhardt et al. described the mode of onset of spontaneous arrhythmias in patients with no structural heart disease, where a single PVC with an extremely short coupling interval (R-on-T phenomenon) triggered a rapid PMVT or VF.^1^ These findings were confirmed by Viskin et al. in a cohort of unexplained cardiac arrest survivors; in the 22 VF episodes recorded, the PVC initiating a rapid PMVT had a coupling interval of 300 ± 52 ms.^2^ Besides, Haïssaguerre et al. found that an early repolarization pattern was present in 31% of 206 patients with IVF; moreover, the early repolarization pattern was associated with a higher risk of recurrent VF.^3^

### Therapeutic management of idiopathic ventricular fibrillation

It is estimated that among out-of-hospital cardiac arrest survivors, IVF is responsible for 5% to 10% of cases.^4,5^ Because recurrent VF events are frequent and poorly predictable, both guidelines and expert consensus documents agree that placement of an implantable cardioverter-defibrillator (ICD) is considered a first-line therapy for IVF patients.^6^ Radiofrequency ablation may be considered when an ICD implantation is contraindicated or refused.^7^ Nevertheless, the role of medical therapy in the treatment of idiopathic ventricular tachycardia (IVT) and IVF is not well established. Current medications in use include isoproterenol, β-blockers, verapamil, and quinidine.^7,8^ However, no reports or studies have described the effectiveness of dopamine in treating this arrhythmia.

#### Pharmacological therapy

Only a few case series studies have described the antiarrhythmic agents’ response and their efficacy in treating IVT/IVF triggered by short-coupled PVCs. The very first few patients diagnosed with IVF were treated with quinidine, and VF was no longer inducible after the quinidine therapy.^9^ The antiarrhythmic action of this drug is attributed to the inhibition of potassium currents, causing a prolongation of action potential duration and refractory periods. It also blocks the fast sodium channels, slowing phase 0 of the action potential and depressing the depolarization phase. Its efficacy is due to the negative dromotropic effect, rendering the myocytes unexcitable by short-coupled PVCs, thereby preventing idiopathic PMVT (IPMVT)/IVF.

Another drug used to reverse PMVT is isoproterenol.^7,10^ In the study by Leenhardt et al., isoproterenol and atropine had mixed effects on suppressing short-coupled PVCs, while verapamil suppressed arrhythmia recurrence in seven out of 12 subjects during their follow-up.^1^ Isoproterenol increases the heart rate due to its β1-/β2-adrenoceptor agonist actions, which increases intracellular calcium concentration, resulting in a steeper slope of the cardiac pacemaker action potential phase 4, and consequently a decrease in basic cycle length. This shortens the QT interval and effective refractory period,^10,11^ thus preventing the excitation of myocytes by the coupled PVCs. On the other hand, the proposed mechanisms of action of verapamil in the treatment of short-coupled ventricular arrhythmias are increasing the refractoriness and coupling intervals, in addition to suppressing the PVCs.

Intravenous amiodarone is also used in the treatment of ventricular arrhythmias. During administration, it blocks the fast sodium channels. It also inhibits norepinephrine release and blocks L-type calcium channels.^12,13^ In the study conducted by Levine et al., amiodarone prevented recurrent episodes of VT/VF in 46% of the patients.^14^

#### Implantable cardioverter-defibrillation

An ICD is the preferred treatment modality in patients with IPMVT/IVF. Trials have demonstrated an improved outcome in patients with ICDs as compared to pharmacological therapy alone.^15,16^ Therefore, it is considered the treatment of choice in patients with IVF. However, given that the ICD does not prevent arrhythmias, adjunctive antiarrhythmic drugs are offered to patients with frequent ICD discharges.

#### Radiofrequency ablation

In patients with IVF, ablation may be considered if the PVCs have a uniform morphology in conjunction with an ICD or when an ICD implantation is refused or contraindicated. Aizawa et al. attempted the first successful IVF ablation in 1992,^17^ whereas Haïssaguerre et al. reported the first series.^18^ Out of 27 patients studied after being resuscitated from recurrent episodes of IVF, 24 (89%) patients had no recurrent VF episodes during the follow-up period. However, at the moment, it is hard to adopt this curative option as the etiology of such a disease is not fully understood.

The patient presented in this report had an electrical storm refractory to amiodarone and lidocaine. After a negative cardiac workup, she was diagnosed with IPMVT/IVF caused by the R-on-T phenomenon, with a coupling interval of 300 ms. Intravenous verapamil boluses were then administered in an attempt to suppress the storm.^8^ However, it failed, besides override ventricular pacing, to terminate the IVF storm.

Due to the lack of isoproterenol, dopamine infusion was started and titrated up to a heart rate of 110 bpm. Of note, only after the heart rate was increased to more than 110 bpm, the IVF storm ceased despite the continued PVC. This may be relevant as it could be suggested to use atrial pacing in such patients to prevent the IVF storm.

Dopamine is a natural precursor of catecholamines. It has both α-and β-adrenergic effects, causing an increase in peripheral vasoconstriction and heart rate.^19,20^ Cardiac β-1 receptors are G protein–coupled receptors. After dopamine binds to the receptor, G_S_-adenylyl cyclase–cyclic adenosine monophosphate–protein kinase-A signaling cascade is initiated, leading to an increase in intracellular calcium influx. An increased intracellular calcium concentration causes a positive ionotropic effect.^20^ In cardiac pacemaker cells, this increase in intracellular calcium causes an increase in the slope of phase 4 of the action potential, similar to that observed during the isoproterenol infusion. Consequently, the pacemaker cells will reach the firing threshold faster. This explains the positive chronotropic effects of dopamine. Therefore, theoretically, both isoproterenol and dopamine have the same effect in terminating IVF.

Accordingly, dopamine successfully terminated the IVF storm in the patient presented in this report despite the ongoing PVCs, probably by affecting repolarization, thereby shortening the QT interval to prevent VF induction. Thereafter, the patient was started on 240 mg of verapamil through a nasogastric tube to control the PVCs and prevent the PMVT/VF recurrence. During the following three days, dopamine was tapered down, and verapamil was continued, after which the burden of PVCs significantly decreased.

Before discharging home, a dual-chamber defibrillator was implanted, and the patient was permanently prescribed 240 mg of verapamil to be taken orally. Eight months of follow-up revealed a complete resolution of IPMVT/IVF and only a few PVCs.

The aim of presenting this case report is to suggest dopamine as a more cost-effective and affordable alternative for isoproterenol. Besides, preparation, administration, and titration of dopamine are more manageable compared to those of isoproterenol. It may also hint at the possibility of using atrial pacing (ventricular pacing may derange the normal myocardial depolarization/repolarization) to increase the heart rate in an attempt to control the IVF storm.

## Conclusion

The use of dopamine in managing IPMVT/IVF has not been described in the literature, although this report is proof of its efficacy. More extensive studies should be conducted regarding its effectiveness in treating such arrhythmias.
